# Evaluation of the adverse drug reaction surveillance system Kadoma City, Zimbabwe 2015

**DOI:** 10.11604/pamj.2017.27.55.11090

**Published:** 2017-05-24

**Authors:** Caroline Muringazuva, Daniel Chirundu, More Mungati, Gerald Shambira, Notion Gombe, Donewell Bangure, Tsitsi Juru, Mufuta Tshimanga

**Affiliations:** 1 Department of Community Medicine, University of Zimbabwe; 2 City Health Department, Kadoma City Council

**Keywords:** Adverse drug reactions, surveillance system, Kadoma, Zimbabwe

## Abstract

**Introduction:**

Medicines have the potential to cause adverse drug reactions and because of this Zimbabwe monitor reactions to medicines through the Adverse Drug Reaction Surveillance System. The Medicines Control Authority of Zimbabwe monitors reactions to medicines through the Adverse Drugs Reactions Surveillance System. The system relies on health professionals to report adverse drug reactions to maximize patient safety. We report results of an evaluation of the Adverse Drugs Reactions Surveillance System in Kadoma District.

**Methods:**

A descriptive cross-sectional study was conducted using the updated CDC guidelines in six health facilities in Kadoma City. Data were collected using a pretested interviewer administered questionnaire, checklists and records review. Data was analyzed using Epi Info^TM^ to calculate frequencies and means. Qualitative data were analyzed manually. Written informed consent was obtained from all study participants.

**Results:**

The surveillance system did not meet up to its objectives as it failed to detect the adverse drug reactions and there was no monitoring of increases in known events. Fewer than half (43%) of the participants were aware of at least 2 objectives of the surveillance system but 83% of health workers willing to participate. However the system was not acceptable, 79% did not perceive the system to be necessary with the majority saying ''why should we fill in the forms when the reactions were already known or minor''. Though the system was supposed to identify potential patient risk factors for particular types of events health workers were reluctant to participate as evidenced by only one form filled out of 20 reactions experienced in the district. The system was simple as the notification form has 16 fields which require easily obtainable information from the patient records.

**Conclusion:**

The surveillance system was not useful and was not acceptable to health workers but was simple and stable. Health workers lacked knowledge. Sharing of results with the Medicines Control Authority of Zimbabwe through the Matrons facilitated training of health workers in Kadoma City. Health workers were encouraged to notify any drug reaction and to completely fill in the notification forms. Patients were also encouraged to report any drug reaction to health care workers.

## Introduction

**Figure 1:**
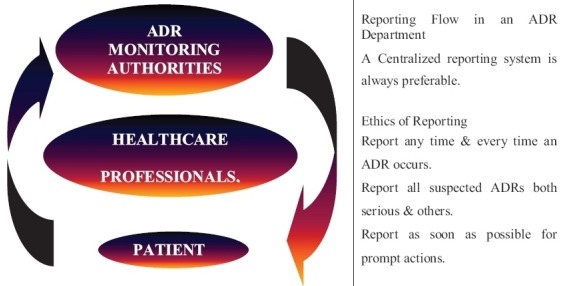
Operation of the ADRSS

All medicines used in clinical practice have the potential to cause adverse drug reactions which can be defined as harmful and unintended responses to a medicine or vaccine. In Zimbabwe this is monitored through the Adverse Drug Reaction Surveillance System (ADRSS) which has the following objectives: to detect new, unusual or rare adverse drug reactions; to monitor increases in known events; to identify drugs with increased number of reported events; to assess the safety of newly licensed drugs. This system is monitored by the Medicines Control Authority of Zimbabwe (MCAZ) with heavy reliance on health professionals to report adverse drug reactions to maximize patient safety [1]. Accumulating evidence suggests that the willingness of healthcare workers to participate in the ADRSS significantly determines reporting rates. Adverse drug reactions can range from minor discomfort to serious harm. A minority can result in fatal complications thus all suspected reactions that occur should be reported in Zimbabwe. MCAZ monitors the safety profile of all medicines ensuring their overall balance of benefits and risks through pharmacovigilance. The outcome of an Adverse Drug Reaction (ADR) can be serious enough to result in illness, injury or even death. ADRs are responsible for a significant number of reported hospital admissions ranging from 0.3% to 7% [2]. Continuous post-marketing monitoring and reporting of ADRs is necessary. Voluntary reporting of ADRs has, for years has been assumed to be the most cost effective and practical way of gathering post-marketing drug safety. Information obtained through voluntary reporting may be limited in representativeness and data quality studies. Studies suggest that the voluntary reporting system may be the only affordable mechanism available for early detection of ADR when they occur. Though this has been assumed to be the most effective way some studies have proved this to be leading to underreporting of ADR's. They found adverse drug events to be underreported in the administrative data of two large university hospitals, despite using an extensive list of ICD-10 codes. Even when set of ICD-10 codes were used to include diagnoses this identified less than 50% of adverse drug reactions [3]. The ability of an ADR monitoring system to help prevent drug-induced injury depends on three factors: there must be a high probability that adverse drug effects will be identified and reported; reports must be reviewed and validated by experts; review results must be fed back to the relevant parties and appropriate regulatory action must be taken. This diagram shows how the system should operate Figure 1.

The Adverse Drug Reaction Surveillance System (ADRSS) is a national drug safety surveillance program sponsored by the Centers for Disease Control and Prevention (CDC). It is a post-marketing safety surveillance program that collects information about ADRs. The system reaffirms the need for early action in regard to the rapid dissemination of information on ADRs. We should be worried about drug reactions because they can cause hospitalisation. If drug reactions are reported this can make a significant difference in the safety of a drug after it has been approved for use. The Zimbabwe ADRSS was introduced 1994. All health care workers are required to report all suspected adverse reactions to drugs including vaccines, x-ray contrast media and complementary medicines especially where the reaction is unusual, potentially serious or clinically significant. This information is disseminated through published quarterly drug and toxicology bulletins. In Zimbabwe there has been a very slow response from health workers in reporting ADRs [1]. The primary objectives of the surveillance system are to: detect new, unusual or rare adverse drug reactions; monitor increases in known events; identify potential patient risk factor for particular types of events; identify drug lots with increased number or types of reported events; assess the safety of newly licensed drugs. In case of an ADR Local Case Report Forms (CRF) should be obtained by the reporter and all sections are to be filled in. The form has at least four sections which are patient information, adverse event or product problem, suspected medication and reporter details. The completed Case Report Form should be sent to the next level till it reaches the national centre or to the manufacturer of the suspected product. Health services delivery in Kadoma City is through one general hospital five Council clinics and private clinics/surgeries. Adverse drug reaction hard copy notification forms are completed at health facilities and subsequently physically transported to the province via the district office. After any presentation by a patient suspecting of a reaction after taking medication, a full medicine and medical history should be done by the health professional, usually a nurse on the day. Investigations should start at the earliest time within 24 hours by the nurse who notifies the District Nursing Officer and the Pharmacist. The District Nursing Officer sends a copy of the notification form to the Provincial Nursing Officer who then sends to the Medicines Control Authority of Zimbabwe through the. Follow up with the patient should be done and feedback given. We evaluated the ADRSS in Kadoma to assess the system performance and reasons for not notifying on time.

## Methods

A surveillance system evaluation was carried out by the public health officer and health professionals from each health facility over a period of three months using the updated Centre for Disease Control Guidelines for Evaluating Surveillance Systems [4]. Attributes assessed were; simplicity, stability, acceptability and completeness. Quantitative Data were analyzed using Epi-info 7.1.5 [5] and qualitative data were analyzed manually. Attributes assessed were; simplicity, stability, acceptability and completeness. Quantitative Data were analyzed using Epi-info 7.1.5. Qualitative data were analyzed manually. Inclusion criteria were based on a calculated sample size of 47 study participants and six health facilities which had offered Mass Drug Administration (MDA). We proportionally sampled to determine the number of participants to be interviewed from each facility. Health workers who were working on each particular day were recruited in the study and purposive sampling was done to health workers who had participated in the MDA. All six Sisters in Charge responsible for reporting ADR to next level were all included in the study. A Pretested interviewer administered questionnaire was used to collect data from health workers. Data collected included information on health worker knowledge on the adverse drug reactions surveillance system and to assess the attributes of the surveillance system. The outpatient registers were checked for any treated cases during and after drug administration. Reports on the ADRSS and minutes of meetings were reviewed. A checklist was used to assess for the availability of the resources needed for running the ADRSS. Data was entered into Epi Info 2012 (CDC) version 7.1.5. The software was used to calculate frequencies, means and proportions at the 5% level of significance. Permission to carry out this study was obtained from Kadoma City Council and Kadoma General Hospital Institutional Review Boards and Health Studies Office Zimbabwe. Written informed consent was obtained from participants. Confidentiality was assured and maintained throughout the study. Names of the participants were excluded from the questionnaires.

## Results

We interviewed 47 health workers. Registered general nurses constituted 30/47 (64%) of the participants. Median years of experience of the participants was 14 years (Q1=6.5, Q3=23.5). Seventy percent (33/47) of the respondents were females

**Assessment of the systems attributes**: Assessment of the system attributes shows that the system was not acceptable but was simple and stable Table 1.

**Table 1:** assessment of systems attributes

ATTRIBUTE	ASSESSMENT
Simplicity	Simple
Usefulness	Not useful
Data quality	Only one form was available
Acceptability	Not Acceptable
Timeliness	Not on time
Stability	Stable

**Knowledge of health workers of the ADRSS Kadoma 2015**: All the 47 health workers were aware of the existence of the ADRSS and knew one or more objectives of the surveillance system. Of the study participants, 20/47 (43%) mentioned the system detects new, unusual or rare drug reactions and (10/47) 21% knew the system could help identify drugs/medicines with increases types of reported events.

**Timeliness of reporting ADRSS Kadoma 2015**: Thirty eight percent of the health workers thought there was no specified time period to notify the ADR, 34% (16/47) indicated that it needs seven days to notify whilst 9/47 (19%) thought this should be immediately done. The available notification form from Kadoma Hospital was immediately notified and sent to the next level. Only 29/47 (62%) of the respondents knew that the forms are supposed to be sent to the DNO whilst council workers send to the matron. However 2/47 (4%) thought the forms are supposed to be sent directly to the PNO or the DHIO.

**Availability of ADRSS guidelines Kadoma 2015**: Only three of the nine departments at the district hospital and each OPD department of the five council clinics had the guidelines.

**Availability of minutes ADRSS Kadoma 2015**: Of the six health facilities studied one health facility had minutes of previous meeting held. The health facility had taken action based on information from the ADRSS though five cases of ADR were attended to. The out patients department registers from all the health facilities were analysed for the period the mass drug administration was done up to two weeks after the end of the last day of administration. The registers showed that children were attended to with reactions but no notifications were done. Key informant interviews with the nurses who were on duty that time revealed that most thought that since the reactions were known and minor there was no need to notify.

**Acceptability and completeness of the ADRSS Kadoma 2015**: Eighty-three percentage of health workers mentioned that they were willing to participate in the Adverse Drug Reaction Surveillance System and accepted the responsibility of providing data. However 79% (37/47) did not perceive the system to be necessary with the majority saying'' why should we fill in the forms when the reactions are already known or minor'' One form out of 20 reactions was filled hence the perceptions and actions of health care workers revealed their reluctance to participate in the surveillance system.

**Stability of the ADRSS Kadoma 2015**: All the 6 health facilities had the Adverse Drug Reactions notification forms, working telephones, and pens and were never out of stock. Of the 11 departments in the 6 health facilities only four had ADR Guidelines.

**Simplicity of the ADRSS Kadoma 2015**: Of the respondents, 28/47 (60%) spent less than 30 minutes to compile and complete the ADRSS data and 17% (8/47) found it difficult to compile the data. One available ADRSS form from the District hospital had nine unfilled fields out the 16 fields which included likely reason for the reaction, investigator`s name and designation.

**Usefulness of the ADRSS Kadoma 2015**: There was no evidence of the system being useful as there was only one available form. There was also no proof of meetings held and decisions made based on the system.

**Resources needed for ADRSS Kadoma 2015**: Resources such as forms, pens, ADRSS forms and telephones were available at all the facilities studied. The standard operating procedures were not displayed in all the five clinics.

**Reasons for Failure to Report**: Reasons mentioned by respondents for failing to report were: lack of training 37/47 (79%), no need to notify since the Adverse reactions were known and minor 33/47 (70%), no ADRs identified 23/47 (48%), and not sure what to capture 7/47 (14%).

**Limitations**: Availability of one notification made it difficult to assess quality of data.

## Discussion

This study revealed that there was lack of good knowledge on the objectives of the surveillance system by the health care workers as less than half were able to mention at least 2 objectives. This was because nearly three quarters of the participants lacked training of the purpose of the surveillance system. Health worker knowledge of the surveillance system and its functions is a necessity for achieving its desired goals and objectives. Hazel et al (2006'6) reported low knowledge levels among the health workers on Adverse Drug Reactions contributed to the underreporting the adverse drug reactions in the United Kingdom [6]. Health care workers` perceptions and actions revealed reluctance to participate in this surveillance system. The willingness of health care workers to participate enables the system to provide accurate, complete, consistent and timely data. The proportion of respondents who knew at least two objectives of the ADRSS was low. This is consistent with a study carried out in Zimbabwe by Khoza S et al. (2004) who found knowledge levels in reporting adverse reactions to be low. Even if some were aware of the reporting channel knowledge on timeliness was generally very low [7]. In 2003 Paul Gavaza et al found out that lack of knowledge and awareness emerged as important factors associated with poor reporting of ADRs by health workers [8]. Stability and simplicity were the strongest components of this surveillance system. The simplicity of the system affects the amount of resources required to operate the system while lack of resources can affect the stability of the system, hence it is easy to stabilise a simple system as is the case in this study.

Usefulness of a system is dependent on its attributes. The ADRSS in Kadoma failed to meet its objectives as a result of under reporting, poor knowledge of health care workers and their unwillingness to participate in this surveillance system. The study sought to ascertain health workers knowledge on ADRSS in Kadoma. The majority were nurses because of their primary importance in the care of patients. In countries where nurses are already participating in the ADRSS, studies have shown that they indeed contribute positively towards the promotion of ADR reporting [9]. In this study we found out that more than half of the respondents were nurses but reporting of the ADR was low. The low reporting rate in Kadoma City is consistent with the findings by Lopez-Gonzalez et al (2009). In that study, under-reporting rate of ADR was very low [10]. The research also provided evidence of a higher reporting of more serious or severe ADRs compared to minor reactions. There is therefore need to improve the reporting of ADRs since they can lead to morbidity and mortality. Under-reporting of ADRs is a worldwide phenomenon and this has been established from previous studies. The determinants of under-reporting, from our study, included perceptions that the reactions were not serious and lack of knowledge of how to report and this was consistent with the findings by Backstrom et al (2000) [11]. Though all respondents reported to have notification forms at their facilities most of them thought it was not necessary to report unless the patient was admitted. As a result all six health facilities are generally not reporting. This was contrary to the finding of Ortega A et al (2008) where reporting ADRSS was on the increase [12].

## Conclusion

We therefore concluded that the system did not meet up with its objectives. The system was not acceptable though was simple and stable. It was not useful and health care workers knowledge on the system was poor. Health workers thought the system was not really useful since most drug reactions were known.

### What is known about this topic

- Medicines have the potential to cause adverse drug reactions;
- There is a system that can monitor drug reactions;
- Adverse drug reactions can be known if only reported by both patients and health professionals.

### What this study adds

- Health workers (79%) did not perceive adverse drug reaction system to be necessary especially if the reactions are already known;
- Most drug reactions are noted during mass drug administrations;
- Awareness to health workers of the need to notify drug reactions can increase reporting of both known and unknown drug reactions.

## Competing interests

The authors declare no competing interests.
